# An integrated AI-enabled system using One Class Twin Cross Learning for early gastric cancer detection

**DOI:** 10.3389/fonc.2025.1623394

**Published:** 2026-01-19

**Authors:** Xian-Xian Liu, Yuanyuan Wei, Yongze Guo, Hongwei Zhang, Huicong Dong, Qun Song, Qi Zhao, Wei Luo, Feng Tian, Juntao Gao, Jiang Cai, Simon Fong, Mingkun Xu

**Affiliations:** 1 Guangdong Institute of Intelligence Science and Technology, Zhuhai, China; 2 Department of Computer and Information Science, University of Macau, Macao, Macao SAR, China; 3 Department of Biomedical Engineering, The Chinese University of Hong Kong, Hong Kong, Hong Kong SAR, China; 4 Department of Neurology, David Geffen School of Medicine, University of California, Los Angeles, Los Angeles, CA, United States; 5 Department of Gastroenterology, Affiliated Hospital of Hebei University of Engineering, Handan, China; 6 Institute of Artificial Intelligence, Chongqing Technology and Business University, Chongqing, China; 7 Cancer Centre, Institute of Translational Medicine, Faculty of Health Sciences, University of Macau, Macao, Macao SAR, China; 8 Ministry of Education (MoE) Frontiers Science Center for Precision Oncology, University of Macau, Macao, Macao SAR, China; 9 The director of the Institute of Clinical Medicine, The First People’s Hospital of Foshan, Guangzhou, China; 10 Hebei Key Laboratory of Medical Data Science, Institute of Biomedical Informatics, School of Medicine, Hebei University of Engineering, Handan, Hebei, China; 11 The Beijing National Research Center for Information Science and Technology (BNRist), Tsinghua University, Beijing, China

**Keywords:** early gastric cancer (EGC), One Class Twin Cross Learning (OCT-X), precision diagnostics, artificial intelligence (AI), computer-aided detection (CAD)

## Abstract

**Background:**

Early detection of gastric cancer, a leading cause of cancer-related mortality worldwide, remains significantly hampered by the limitations of current diagnostic technologies, resulting in high rates of misdiagnosis and missed diagnoses.

**Methods:**

To address these clinical challenges, we propose an integrated AI-enabled imaging system that synergizes advanced hardware and software technologies to optimize both speed and diagnostic accuracy. Central to this system is our newly developed One Class Twin Cross Learning (OCT-X) algorithm, which leverages a fast double-threshold grid search strategy (FDT-GS) and a patch-based deep fully convolutional network for precise lesion surveillance and classification in real-time. The hardware platform incorporates an all-in-one point-of-care testing (POCT) device, equipped with high-resolution imaging sensors, real-time data processing capabilities, and wireless connectivity, supported by the NI CompactDAQ system and LabVIEW software for seamless data acquisition and control.

**Results:**

This integrated system achieved a diagnostic accuracy of 99.70%, outperforming existing state-of-the-art models by up to 4.47%, and demonstrated a 10% improvement in multirate adaptability, ensuring robust performance across varied imaging conditions and patients profiles.

**Conclusion:**

These results highlight the potential of the OCT-X algorithm and the integrated platform to enable more accurate, efficient, and non-invasive early detection of gastric cancer in point-of-care settings.

## 1 Introduction

According to GLOBOCAN 2022 statistics Bray et al. (1), gastric cancer remains a major global health concern, ranking as the fifth most common malignancy and the fifth leading cause of cancer-related mortality worldwide. Early detection is crucial for improving patient outcomes; however, it presents substantial challenges due to the subtle symptoms and non-specific symptoms associated with early-stage disease, alongside difficulties in distinguishing early gastric cancer (EGC) lesions from benign conditions Zhai (2). Furthermore, the scarcity and limited representativeness of EGC datasets often result in insufficient analytical capability and diagnostic accuracy Ansari et al. (3). Compounding these issues, imbalanced class distributions - particularly the preponderance of unlabeled negative cases-introduce bias and reduce reliability of conventional machine learning models Ul Haq et al. (4).

A range of studies has contributed to the development of computer-aided diagnosis (CAD) systems for EGC detection. Early work by Mizumoto et al. (5) combined artificial intelligence (AI) with magnifying endoscopy with narrow band imaging (ME-NBI), achieving enhanced diagnostic precision. Muto et al. (6) developed a CAD framework based on magnifying narrow-band imaging (M-NBI) with high sensitivity and specificity, while Wang et al. (7) proposed a CAD system leveraging double contrast-enhanced endoscopic ultrasonography (DCEUS). Additionally, Osawa et al. (8) investigated the application of flexible spectral imaging color enhancement for EGC detection. Beyond endoscopic imaging, alternative modalities such as computed tomography (CT) Teng et al. (9), endoscopic images Ma et al. (10), double contrast enhanced ultrasonography Urakawa et al. (11), CT radiomics analysis Wu et al. (12), optical chromoendoscopy Saito (13), linked color imaging Umegaki et al. (14), confocal laser endomicroscopy Cho et al. (15), and auto-fluorescence imaging Chen et al. (16) have been explored for computer-aided EGC diagnosis.

Although conventional diagnostic technologies like endoscopy and biopsy are valuable for evaluating tumor depth in EGC, they have inherent limitations. They are invasive, potentially uncomfortable and reliant on subjective human interpretation, leading to diagnostic uncertainty Olsson et al. (17) and undiagnosed illness. To overcome these limitations, there is a pressing need for rapid, accurate, low-cost, and less invasive diagnostic tools. Wireless endoscopy capsule monitoring system offers a promising alternative, allowing for non-invasive gastrointestinal tract navigation, with image acquisition and transmission facilitated by advanced systems such as NI CompactDAQ, as well as minimally invasive gastric surgery-especially for complex procedures such as lymphadenectomyMarano et al. (18). These innovations represent a significant advancement toward more precise and patient-friendly diagnostic and therapeutic approaches.

In recent years, the advent of AI-driven applications has catalyzed transformative advances in gastrointestinal diagnostics in clinical practice. Machine learning and deep learning algorithms have notably enhanced diagnostic accuracy, reduced invasiveness, and minimized human error. Notable contributions include Rajeswari et al. (19) demonstrated that the MIFNET deep learning algorithm enhances gastric cancer detection accuracy through a novel classification method incorporating histological features, genotypes, and genetic phenotypes. Meanwhile, Zhang et al. (20) showed that the IMR-CNN model excels in EGC detection with precision and recall rates of 92.9% and 95.3%, while Chae and Cho (21) developed a computer-aided system utilizing the Vision Transformer model with Multi-Filter AutoAugment achieved F1 scores of 0.87 for identifying abnormalities and 0.92 for distinguishing EGC. Additional approaches include MPCs for tumor size-agnostic feature extraction introduced by Padthe et al. (22), hybrid CNN-RNN classification methods proposed by Prince et al. (23), and Hybrid Deep Learning Models that aid endoscopists in diagnosing esophageal tumors with recognition rates of 97.81%. Srivastava et al. (24) who utilized convolutional neural networks (CNNs) to achieve high accuracy in detecting early-stage gastric cancer from endoscopic images. Similarly, Jamil et al. (25) developed a machine learning model that integrates clinical data with imaging features, resulting in improved predictive performance for EGC prognosis. Yalamarthi et al. (26)explored the use of AI in automating the detection process, which significantly reduced the time required for diagnosis and minimized human error. Macdonald et al. (27) focused on the application of machine learning algorithms to differentiate between malignant and benign gastric lesions, showcasing how AI can assist in making more accurate clinical decisions and potentially reduce the number of unnecessary biopsies. Moreover, Ikenoyama et al. (28) explored the integration of convolutional neural networks (CNNs) to enhance the accuracy of endoscopic image analysis, thereby improving the detection rate of early lesions. Yoon et al. (29) focused on implementing deep learning models to differentiate between benign and malignant gastric conditions with high precision, which aids in reducing false positives and negatives. Tang et al. (30) developed a comprehensive AI framework that combines multiple machine learning algorithms to analyze heterogeneous datasets, addressing issues related to data variability and scarcity. Wu et al. (31) introduced advanced data augmentation techniques to mitigate the effects of limited dataset sizes, thereby enhancing the robustness and generalizability of diagnostic models. Shibata et al. (32) tackled the problem of imbalanced class distribution by employing novel sampling methods and ensemble learning techniques, which help in balancing the dataset and minimizing biases in the diagnostic process.

While these AI approaches show promise, they face significant challenges when deployed in real-time, embedded diagnostic systems—particularly in handling extreme class imbalance and leveraging unlabeled data. In clinical settings, EGC cases are rare compared to normal or benign findings, leading to severely imbalanced datasets. Furthermore, the vast majority of collected data are unlabeled, posing a major obstacle for conventional supervised learning methods. Although one-class classification methods offer potential solutions, current approaches have limitations in embedded real-time applications. Goyal et al. (33) proposed the Deep Robust One-Class Classification(DROCC), which is used in a one class problem that do not require any auxiliary information in various detection domains, and it is acknowledged that detecting abnormal positive is robust. Empirical assessment has proved that DROCC is very effective on the settings of two different types of One-Class problems and the actual data sets in a series of different fields: table data, image (CIFAR and ImageNet), audio and time sequences, which can increase up to 20% of accuracy in terms of abnormal detection. The One-Class Support Vector Machine (OC-SVM) proposed by Shahid et al. (34) is a widely used approach to one class classification, the problem of distinguishing one class of data from the rest of the feature space. Its main advantage is to train the classifier using only patterns belonging to the target class distribution. The OC-SVM is effective when large samples are available for providing an accurate classification. Sun et al. (35) introduced a novel end-to-end model that integrates the One-Class Support Vector Machine into Convolutional Neural Network (CNN), named Deep One-Class (DOC) model. Specifically, the robust loss function derived from the one-class SVM is proposed to optimize the parameters of this model. Compared with the hierarchical models, the DOC model not only simplifies the complexity of the process, but also obtains the global optimal solution of the whole process. As for semi-supervised learning, Yessoufou and Zhu (36) used a one-class convolutional neural network (OC-CNN) model. The OC-CNN model combines a one-class (OC) classification algorithm with a simple one-dimensional convolutional neural network (1D CNN) configuration. Using the prediction error loss of the proposed OC-CNN model as an ideal positive-sensitive feature for rapid positive detection. La Grassa et al. (37) developed a novel model named One Class Minimum Spanning Tree (OCmst) for the novelty detection problem. This model utilizes a Convolutional Neural Network (CNN) as a deep feature extractor and a graph-based approach built on the Minimum Spanning Tree (MST). The training data remains unpolluted by outliers (abnormal class), aiming to accurately discern whether a test instance pertains to the normal class or the abnormal class. These advancements collectively represent significant strides in overcoming the inherent challenges in EGC diagnostics through the application of sophisticated AI methodologies.

To address these specific challenges, we propose an integrated AI-enabled system using a novel One Class Twin Cross Learning (OCT-X) algorithm for early gastric cancer detection. The OCT-X framework is specifically designed to handle extreme class imbalance and leverage unlabeled data in real-time, embedded systems for EGC detection. It introduces a unique fast double threshold search strategy for effective preprocessing, distinguishing between potential and noise patches within endoscopic images. A patch-based deep fully convolutional network, integrated with LabVIEW multirate algorithm and NI CompactDAQ for real-time data acquisition, enhances both detection accuracy and operational efficiency while addressing the core challenges of imbalanced and unlabeled data in embedded environments.

The contributions of our work are as follows:

1. Addressing extreme class imbalance and unlabeled data: The OCT-X algorithm specifically targets the challenges of extreme class imbalance and unlabeled data in real-time embedded systems for EGC detection. By employing a novel twin cross learning architecture, our method effectively leverages limited labeled EGC data while utilizing abundant unlabeled data, achieving an average AUC of 93.13% across the four datasets (GU, GRS, GPs, and GB). This represents an improvement in average performance compared to existing methods, with relative increases of 4.96% over DROCC, 18.65% over OC-SVM, 15.72% over DOC, and 14.42% over HDLM (XGBoost). The performance gap was most notable on the GRS dataset, where our model showed a 9.19% higher AUC than the next best method. These findings suggest that the proposed approach offers a promising alternative for tasks in this domain.
2. Real-time embedded system optimization: Our framework is specifically designed for deployment in embedded clinical systems using NI CompactDAQ and LabVIEW multirate processing. The OCT-X algorithm incorporates computationally efficient strategies that enable real-time operation while maintaining high accuracy, addressing the critical gap between algorithmic performance and practical embedded implementation for EGC diagnostics.
3. Advancement in multirate learning for imbalanced data: By incorporating a multirate learning mechanism integrated with LabVIEW’s multirate parallel processing, the OCT-X algorithm effectively addresses biases associated with unbalanced sample learning in real-time scenarios. This approach enables simultaneous processing of data at multiple resolutions and rates, particularly beneficial for handling the extreme class imbalance characteristic of EGC datasets.
4. Clinical impact in resource-constrained environments: The OCT-X framework demonstrates substantial potential as a clinical decision support system for EGC detection in embedded settings. By achieving a 10% improvement in accuracy for multirate adaptability and outperforming contemporary models, this system offers a valuable tool for enhancing clinical diagnostic processes in resource-constrained environments where timely, accurate diagnostics are essential.

## 2 Hardware implementation with add-on NI LabVIEW module on NI CompactDAQ for real-time adaptive modulation schemes

**Figure 1:**
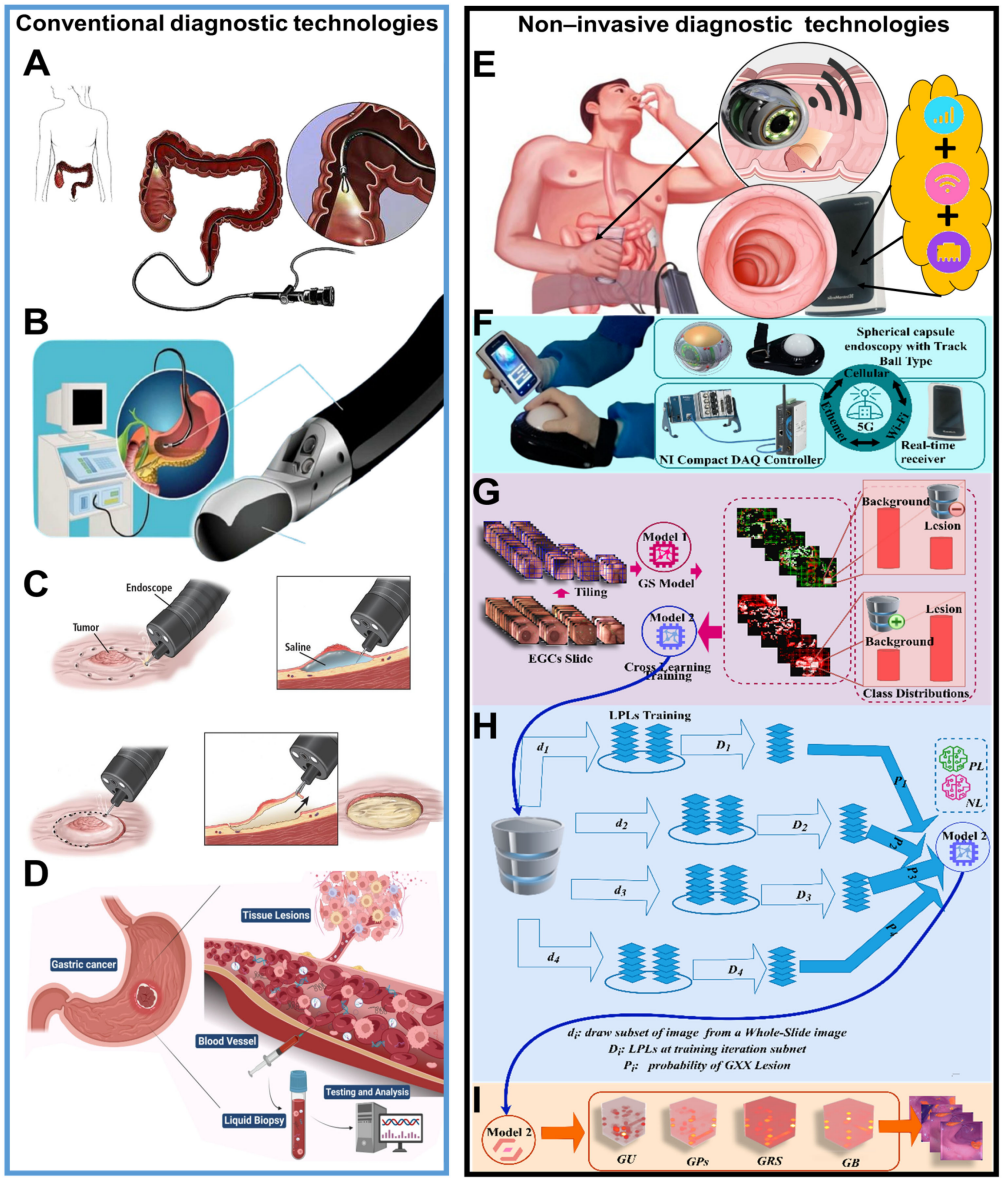
Illustration comparing conventional and FDT-GS driven OCT-X diagnostic technologies. **(A–D)** depict traditional methods like gastrointestinal endoscopy, endoscopic ultrasound (EUS), biopsy with endoscopic submucosal dissection (ESD) and liquid biopsy with blood test (38–40) under CC BY 4.0. **(E–I)** show advanced non-invasive technologies such as integrated NITM enhanced remote capsule monitoring based on multipath signal enhancement, the integration of NI CompactDAQ with LabVIEW software for the FDT-GS model, scalable sub-networks architecture and confidence prediction.

Compared to other open invasive diagnosis like gastrointestinal endoscopy, endoscopy ultrasound, biopsy and liquid biopsy depicted in **Figures 1A–D**, the advanced invasive solution of hardware integration, as illustrated in **Figures 1E, F**, features an all-in-one POCT device tailored for EGC detection. This device combines high-resolution imaging sensors, real-time data processing algorithms (refer to **Figures 1G–I**), and wireless connectivity. The CompactDAQ system integrates hardware for data input/output with NI LabVIEW software, facilitating the collection, processing, and analysis of sensor data. Enhanced by 5G signal transmission via cellular, Ethernet, and Wi-Fi, the system enables real-time lesion surveillance and remote monitoring.

The trackball interface bridges user commands and 5G signals, ensuring seamless wireless transmission to the endoscopy capsule. Cellular networks provide high-speed data transfer and remote control functionalities, while Ethernet ensures secure data processing and communication. Wi-Fi adds flexibility and mobility, enabling wireless data exchange and remote monitoring. This integration allows efficient control of the endoscopy process, real-time data processing, and lesion surveillance. The device’s portability, user-friendly interface, and integration with electronic health records enhance accessibility, usability, and documentation accuracy, ultimately improving detection rates and patient outcomes.

The integration of NI CompactDAQ with LabVIEW enables adaptive modulation techniques to optimize data rates based on varying channel conditions. By combining NI CompactDAQ for data acquisition and LabVIEW for real-time image processing and modulation control, the system dynamically adjusts the modulation scheme to achieve optimal data rates for image transmission in different classes.

The system continuously monitors key parameters such as signal-to-noise ratio (SNR) using NI CompactDAQ hardware. LabVIEW processes this information in real-time, selecting the most suitable modulation scheme for transmitting image data, ensuring reliable communication even in challenging scenarios Costanzo et al. (41). The feedback loop between the real-time receiver and capsule endoscopy video transmitter components allows for dynamic modulation scheme switching based on received channel feedback.

LabVIEW supports multiple modulation schemes. By defining class-specific modulation schemes tailored to the SNR of each object class and utilizing LabVIEW for real-time analysis and modulation selection, the system dynamically adjusts data rates to optimize detection performance. Establishing a feedback loop between the object detection system and the modulation control system enables real-time speed adaptation, allowing seamless switching between predefined modulation schemes based on the movement characteristics of detected objects. This dual modulation scheme approach ensures the system adapts to the speed requirements of different object classes, facilitating efficient channel adaptive cooperative transmission (CACT) for accurate detection across various classes with varying speed profiles.

**Figure 2:**
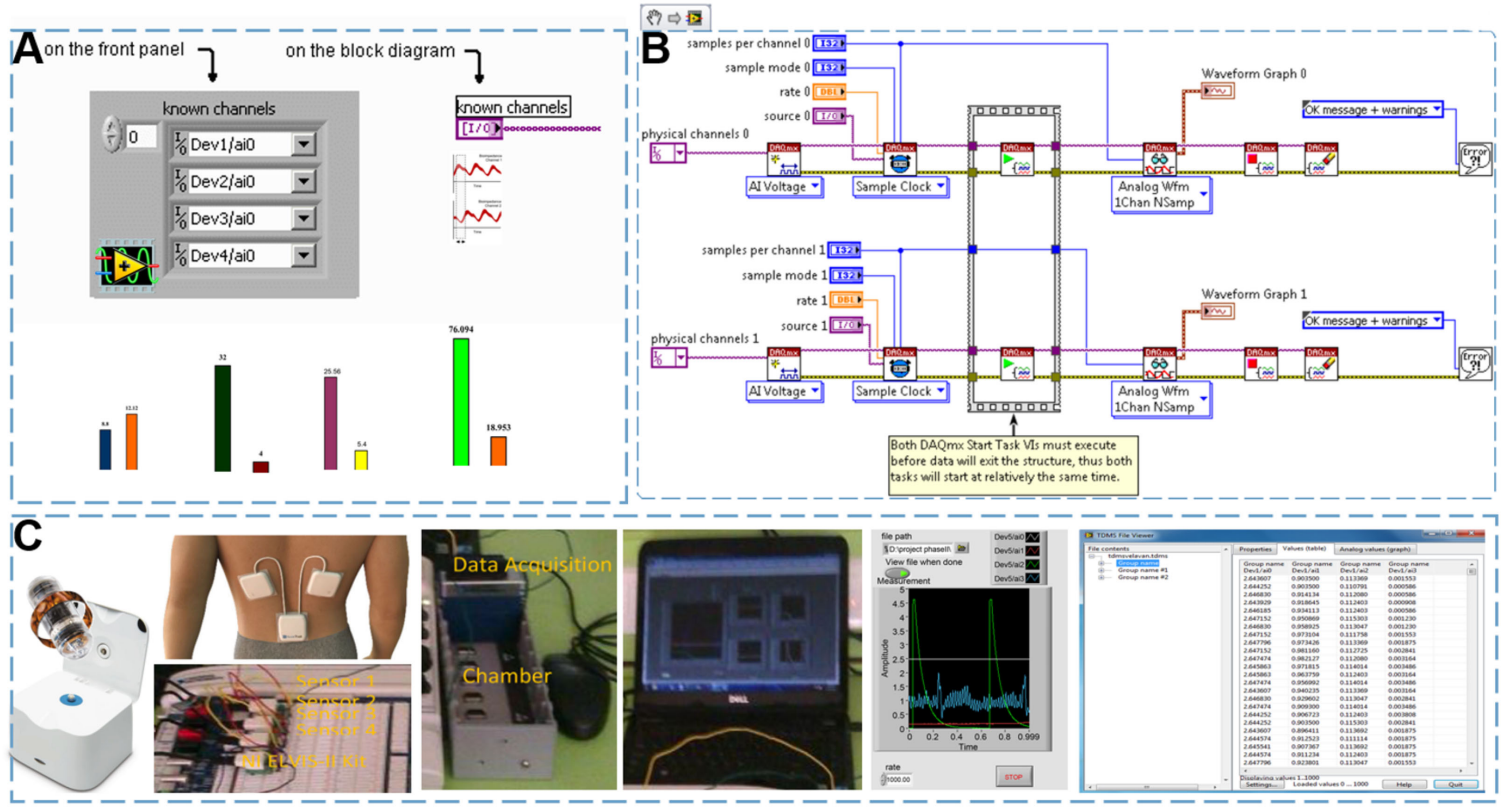
Hardware connection of the multi-rate embedded computing system for EGC detection. **(A)** shows system configuration of multichannel DAQP modules (Dev1-Dev4) with class distribution chart. **(B)** depicts block diagram of NI-DAQmx signal‑acquisition chain and PID control devices. **(C)** includes NI-9237 DAQ hardware components interfaced with LabVIEW for data acquisition and processing.

Advanced API Control with the LabVIEW Control Design and Simulation Module could effectively increase determinism and faster control loops with LabVIEW real-time and LabVIEW FPGA. The **Figure 2A** show the multi-channel data acquisition can be performed using LabVIEW. Raw and post-processed data were analyzed in the frequency domain. This analysis confirmed that both signals were distributed in the same frequency band throughout the entire time without aliasing. The data acquisition NI-DAQ™mx system (DAQ) needs to be capable of recording multisensors simultaneously at rates up to 12.8kS/s/ch. Many models have both a data imbalance and an imbalance rate problem in training model. While negative and positive learning are typically done independently, you can use a PC-based data acquisition system to operate both simultaneously within the LabVIEW ADE. **Figure 2B** shows an ADE PID Control Toolkit of a LabVIEW block diagram for controlling both negative and positive learning. In this block diagram, the measured error value is compared to the threshold, each connected to either the sampling or the replication. In **Figure 2C** show the real-time operating system (RTOS) provides the maximum level of software determinism and reliability for control systems by dedicating all resources to a deployed application. Using the LabVIEW real-time module, you can develop and deploy applications to all NI real-time hardware targets including standard desktop PCs and PXI systems. With the NI-DAQmx driver software, you can easily migrate PCI, PCI Express, and PXI platform devices from LabVIEW for Windows to LabVIEW Real-Time on CompactRIO or PXI and retain the same function calls and hardware configuration.

## 3 Overview of model architecture

**Figure 3:**
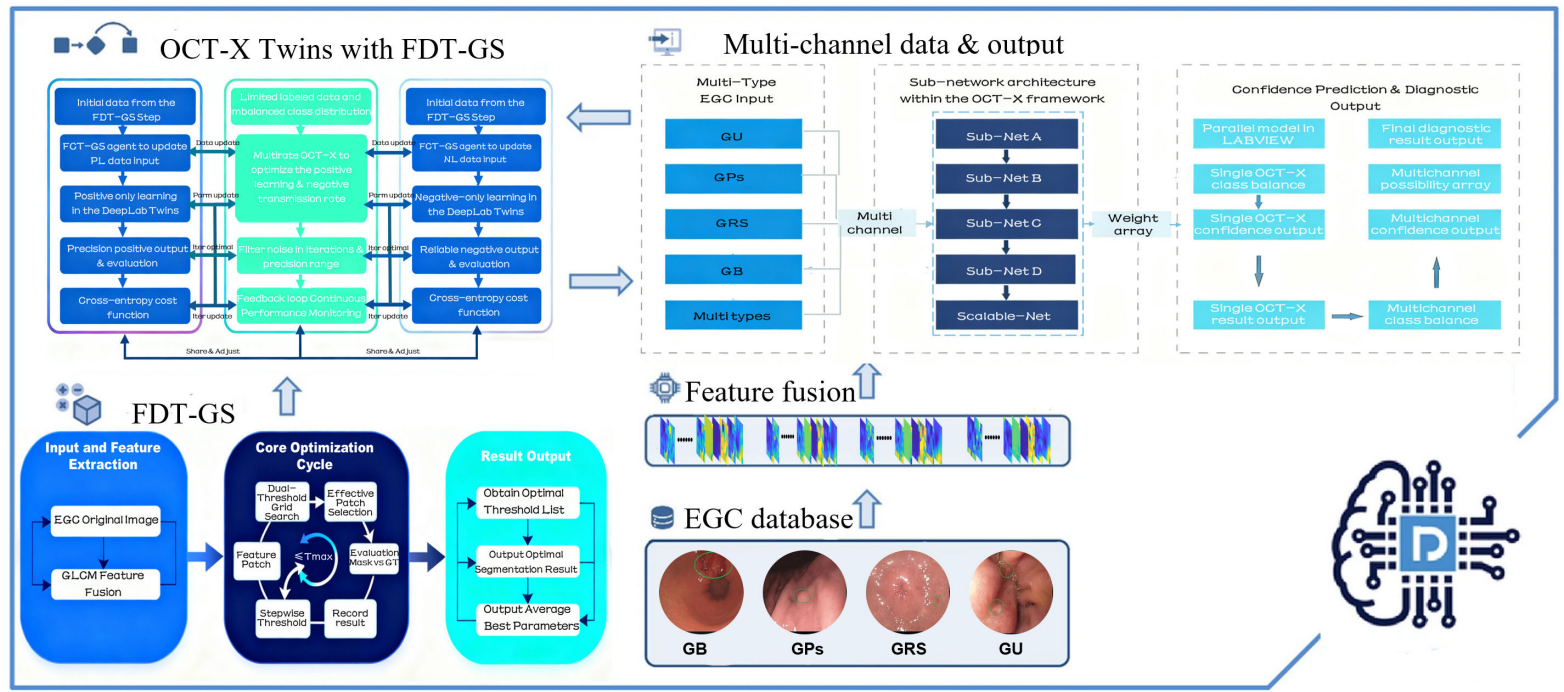
Flowchart of the OCT-X twins with an FDT-GS agent for multi-type patch-based segmentation. The diagram illustrates GLCM feature extraction and fusion, FDT-GS preprocessing, and twins cross learning for scalable lesion segmentation across multi channel gastric data, from raw input through to diagnostic prediction.

The proposed system architecture in **Figure 3** for early gastric cancer detection employs the One-Class Twin Cross Learning (OCT-X) framework, a novel methodology designed to address class imbalance and noisy label challenges in medical image analysis. The system is constructed on a patch-based architecture integrated with Gray-Level Co-occurrence Matrix (GLCM) feature fusion, establishing the foundation for all subsequent analytical processes.

The framework employs a scalable modular network wherein original endoscopic images are systematically partitioned into non-overlapping patches. Each patch then undergoes comprehensive texture characterization through GLCM feature extraction to capture discriminative spatial information of gastric tissue structures.

The core innovation lies in the OCT-X twin-network architecture. Through a specialized cross-learning mechanism, each branch—dedicated to either Positive-Only Learning or Negative-Only Learning—progressively enhances its class-specific discrimination capability. This is achieved by strategically augmenting target-class information patches while simultaneously reducing non target noisy patches through cross-entropy cost optimization. This dual-strategy approach enables progressive refinement of single-class identification performance.

Supporting this learning process is the Fast Double-Threshold Grid Search (FDT-GS) agent, which functions as an auxiliary component. The OCT-X based FDT-GS agent implements a dual-threshold semi-supervised search strategy to dynamically reinforce target-class information patches while filtering out non-target noisy patches. Optimized through a reinforcement learning-inspired reward mechanism guided by F1 score improvements, it contributes to enhanced data purity and feature representation quality for each sub-network.

Processed multi-channel data are subsequently fed into the Multi-channel OCT-X Cross Learning engine, where four specialized sub-networks operate in parallel to execute coordinated positive and negative learning. This twin-cross architecture maintains balanced learning rates across all channels, preventing any single data stream from dominating the training process. The OCT-X based FDT-GS agent continuously supports this procedure through its reward-driven mechanism that monitors performance metrics across all learning paths. By systematically enhancing target information while suppressing noise through our patch-based fully convolutional network, OCT-X effectively delineates the feature space to improve discrimination between subtle early gastric cancer (EGC) lesions and general inflammation conditions, thereby advancing the state-of-the-art in imbalanced medical image analysis.

### 3.1 Software system algorithm implementation

**Figure 4:**
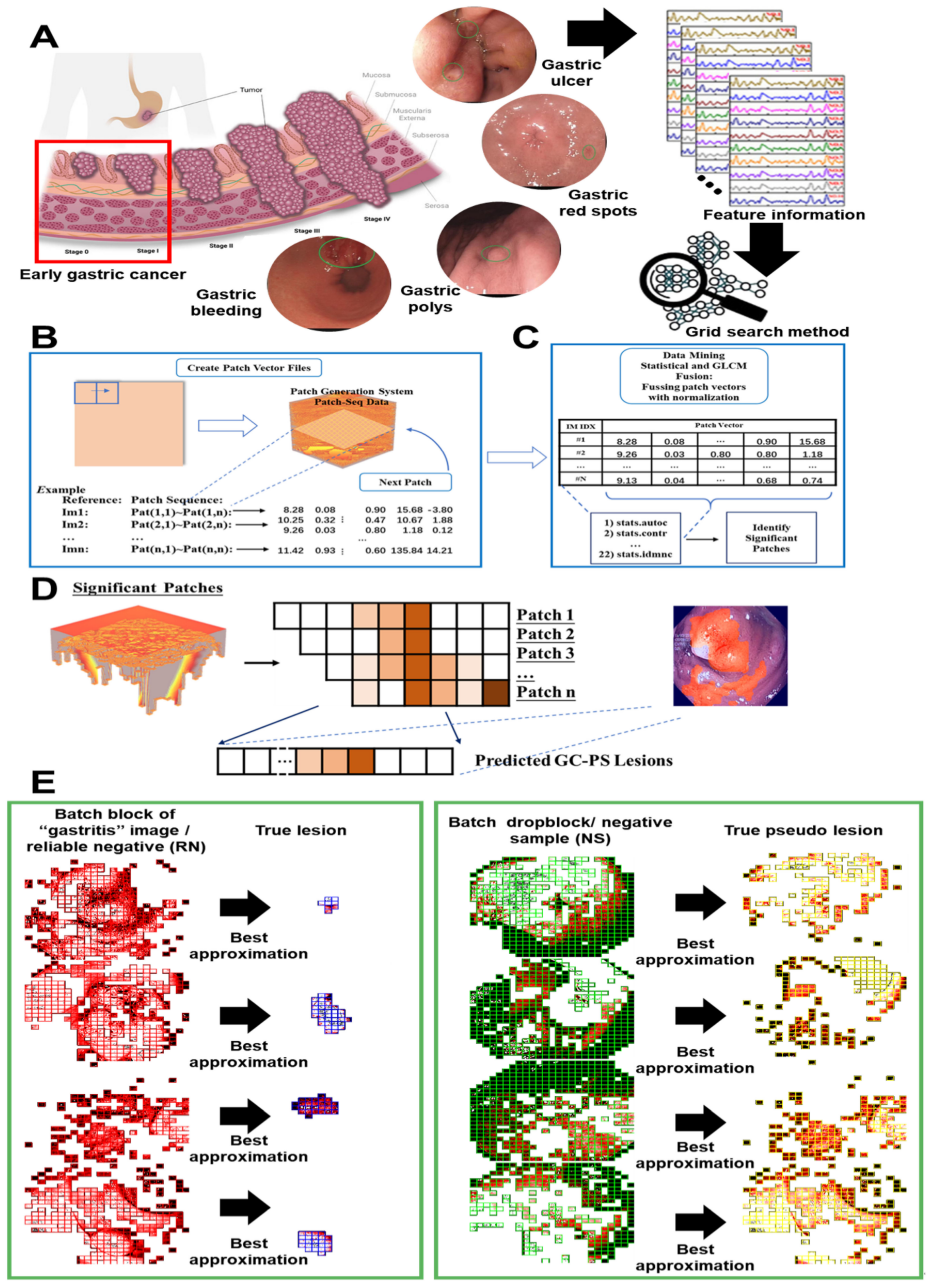
Diagram depicting a process for the FDT-GS retrieval system for EGC detection. **(A)** shows EGC sample type and extracting feature information from GLCM indexes for data preprocessing on FDT-GS. **(B)** details generating patch vector files. **(C)** involves generation and fusion of GLCM maps. **(D)** illustrates identification of significant patches and predicted lesions. **(E)** compares visualization of batch analysis through occupancy‑grid mapping.

The software system implementation, depicted in **Figure 4**, involves trimming the image set based on patching. The FDT-GS model, describes in **Algorithm 1**, selects high-quality negative samples for effective prediction and potential positive patches for positive learning. This approach addresses the issue of imbalanced training samples in the two sub-networks. In the negative-only learning model, the background label predominates over the lesion label, whereas in the positive learning model, the lesion labels are more prevalent than the background labels. This balance solves the problem of imbalanced training samples, which is one of the advantages of our model.

The output of the network (after applying SoftMax) for each transformed image associated with the original one is a vector $\theta =(\theta_{P+},\theta_{P-},\theta_{N+},\theta_{N-})$, where $\theta_{j}$ is the probability of the transformed image to belong to class j∈{P+,P−,N+,N−}. The DeepLab classification model in this study, with the base model (ResNet-50), is trained to predict the patches, where θ represents the probability of the transformed image belonging to one of the new four classes $\left(P_{+},P_{-},N_{+},N_{-}\right)$. We propose an inference process to fuse the output of these two transformed images ( $x_{i}^{+}$ and $x_{i}^{-}$) to predict the label of the original image ( $x_{i}$) based on the Fusion of DeepLab Twins. For each pair $(x_{i}^{+}$ and $x_{i}^{-})$, the prediction of the original image $\left(y_{i}\right)$ will be either P or N. Let $\hat{y_{i}^{+}}=\arg\text{ max }\theta =\arg\text{ max }(\theta_{P+},\theta_{P-},\theta_{N+},\theta_{N-})$ and $\hat{y_{i}^{-}}=\arg\text{ max }\psi =\arg\text{ max }(\psi_{P+},\psi_{P-},\psi_{N+},\psi_{N-})$ be the ResNet-50 predictions for $x_{i}^{+}$ and $x_{i}^{-}$ respectively. The decision rule is given in Equation 1:

If $\hat{y_{i}^{+}}=N+$ and $\hat{y_{i}^{-}}=N-$, then $\hat{y_{i}}=N$.

If $\hat{y_{i}^{+}}=P+$ and $\hat{y_{i}^{-}}=P-$, then $\hat{y_{i}}=P$.

If none of the above applies, then.

(1) $$\hat{y_{i}}=\{\begin{array}{ll} N & \text{if max }(\theta_{Nj},\psi_{Nj})>\text{max }(\theta_{Nj},\psi_{Nj}),\;j\in \{+,-\}\\ P & \text{otherwise}. \end{array}$$

The trained cross-learning model is depicted in **Figure 4C**, where four sub-networks (Sub-Net A, Sub-Net B, Sub-Net C, and Sub-Net D) with the same structure are used in the one-class cross learning model. The model provides an anomaly score via the confidence, referred to as the positive reliability of the prediction. By adding the confidence score, which mathematically corresponds to the probability of whether the data belongs to a positive class or not, the model enhances learning accuracy.

In **Figure 4D**, the 3D EGC feature heatmap based on the patch result is depicted. The heatmap showcases a color-coded representation of the intensity or activation level of the 22 EGC features across the three-dimensional space. The colors on the heatmap range from dark colors (such as gray) indicating lower intensity to bright colors (such as yellow or red) indicating higher intensity. The heatmap visually highlights specific regions or areas within the 3D space where the EGC features are more prominent or concentrated. This information can be valuable in analyzing and interpreting the distribution and significance of these features in the context of the given dataset or study.

### 3.2 Adaptive algorithm procedure

The FDT-GS is conducted as the global processing stage. We perform a FDT-GS with four datasets and conduct an ablation study with the FDT-GS method to investigate potential EGC. The FDT-GS typically identifies a better set of hyperparameters than a manual search within the same amount of time. The OCT-X learning serves as the refinement stage for GC segmentation.

### 3.3 Sample preprocessing and augmentation

We collected and evaluated our method on two datasets donated by Foshan Hospital in 2021. The dataset contains four types of lesions in gastric cancer (GC): gastric ulcer (GU), gastric red spots (GRS), gastric polyps (GPs), and gastric bleeding (GB), as shown in **Figure 4A**. Ground truth annotations were provided by experienced doctors. Due to the large number of frames available (around ten thousand), experts often outline lesions with an elliptical approximation to cover as much of the lesion as possible. Examples of the ground truth superimposed on original frames are provided in this study. All four datasets were partitioned into 50px × 50px patches. The Benign-to Malignant ratios for GU, GRS, GPs, and GB were set to 11:8, 6:28, 32:25, and 50:68, respectively. The resulting patches were divided into training, testing, and validation sets following a 7:2:1 ratio. As detailed in **Table 1**, all four lesion types exhibit class imbalance to varying degrees. Specifically, GRS (17.6% benign vs. 82.4% malignant) and GB (12.4% benign vs. 87.6% malignant) demonstrate severe imbalance, GU (58.4% benign vs. 41.6% malignant) shows mild imbalance, while GPs (56.1% benign vs. 43.9% malignant) remains relatively balanced.

**Table 1:** Dataset statistics information.

Dataset type	Patch size	Class (B/L)	Training set (B/L)	Testing set (B/L)	Validation set (B/L)	Totals	Class distribution	P-value
GU	50px × 50px	11/8	1301/946	371/270	185/135	Total: 1,857 Training: 2,247 Testing: 641 Validation: 320	Benign: 58.4% Malignant: 41.6% (Mild Imbalance)	p < <0.001 **
GRS	50px × 50px	6/28	709/3312	202/946	101/473	Total: 5,743 Training: 4,021 Testing: 1,148 Validation: 574	Benign: 17.6% Malignant: 82.4% (High Imbalance)	p <0.0001 ****
GPs	50px × 50px	32/25	3785/2957	1081/845	540/422	Total: 9,630 Training: 6,742 Testing: 1,926 Validation: 962	Benign: 56.1% Malignant: 43.9% Relatively Balanced)	p <0.01 *
GB	50px × 50px	50/68	118/828	33/236	16/118	Total: 1,349 Training: 946 Testing: 269 Validation: 134	Benign: 12.4% Malignant: 87.6% (High Imbalance)	p <0.0001 ****

B/M: Benign/Malignant counts; p-values from proportion tests (H: 50/50 balance); Significance: *p < 0.05, **p < 0.01, ***p < 0.001, ****p < 0.0001.

Additional preprocessing was conducted using standard procedures. Data augmentation techniques were applied to mitigate the imbalance of samples across different classes and enhance the training of machine learning models Sampath et al. (42). These techniques include rotation, scaling, flipping, and color adjustments, which help in increasing the diversity of the training data and improving the model’s generalization capabilities.

### 3.4 Fast preprocessing model

The proposed pipeline, shown in **Figures 4B–E**, encompasses three main components: preprocessing and preparation of input data, feature extraction, fusion and feature abstraction, and classification segmentation.

1. Preprocessing and feature extraction: In **Figure 4B**, we decompose the gastric endoscopy image into patches to generate the Gray-Level Co-occurrence Matrix (GLCM) value of each patch. Each frame image in the gastroscope video clip is divided into non-overlapping P * P patches. All GC frames were formatted into patch vector files, with patch selection based on predefined conditions (optimized parameters, search spaces, and the number of iterations). Each file contains the patch ID and GLCM measurement of the GC patch under calculation.
2. Fusion and feature abstraction: In **Figure 4C**, ground truth (GT) was applied to the patch in each captured frame as binary 0/1 vectors, correlating the patch features composition to the image as a continuous sequence. We classified embedding GLCM value sensitivity against each patch as lesion or no-lesion corresponding to each image or GLCM search threshold, respectively. FDT-GS average search range was also utilized as a univariate analysis calculating odds-ratio and statistical significance to identify strong associations between the presence or absence of a lesion.
3. Classification segmentation: Data mining methods were applied to all sets of patch vectors, each set corresponding to a particular patch. Patches that included statistically significant patch sites by fusing GLCM maps, normalizing fusing attention, and achieving an overall accuracy of >5% by FDT-GS were marked as significant and considered for further deep learning. In each significant patch, patches with significant correlation with the GC lesion identified were highlighted and stored as GC potential areas impactful for GC prediction.

In **Figure 4D**, significant patches were generated for further machine learning based on the selected GLCM range and evaluated by the evaluation indexes.

In **Figure 4E**, the FDT-GS model outcomes undergo fine-tuning using a combination of authentic lesion and pseudo-lesion (non-cancer) data. Within these datasets, a batch block consisting of “gastritis” patches is identified as the reliable positive (RP), providing a strong representation of the positive class. Simultaneously, the batch drop block is chosen as the negative sample (NS), effectively’ approximating the negative class for optimal learning. This strategic optimization process ensures that the model is trained comprehensively on diverse datasets, thereby enhancing its precision in distinguishing between lesions and non-cancerous conditions.

### 3.5 OCT-X single modal network model

**Figure 5:**
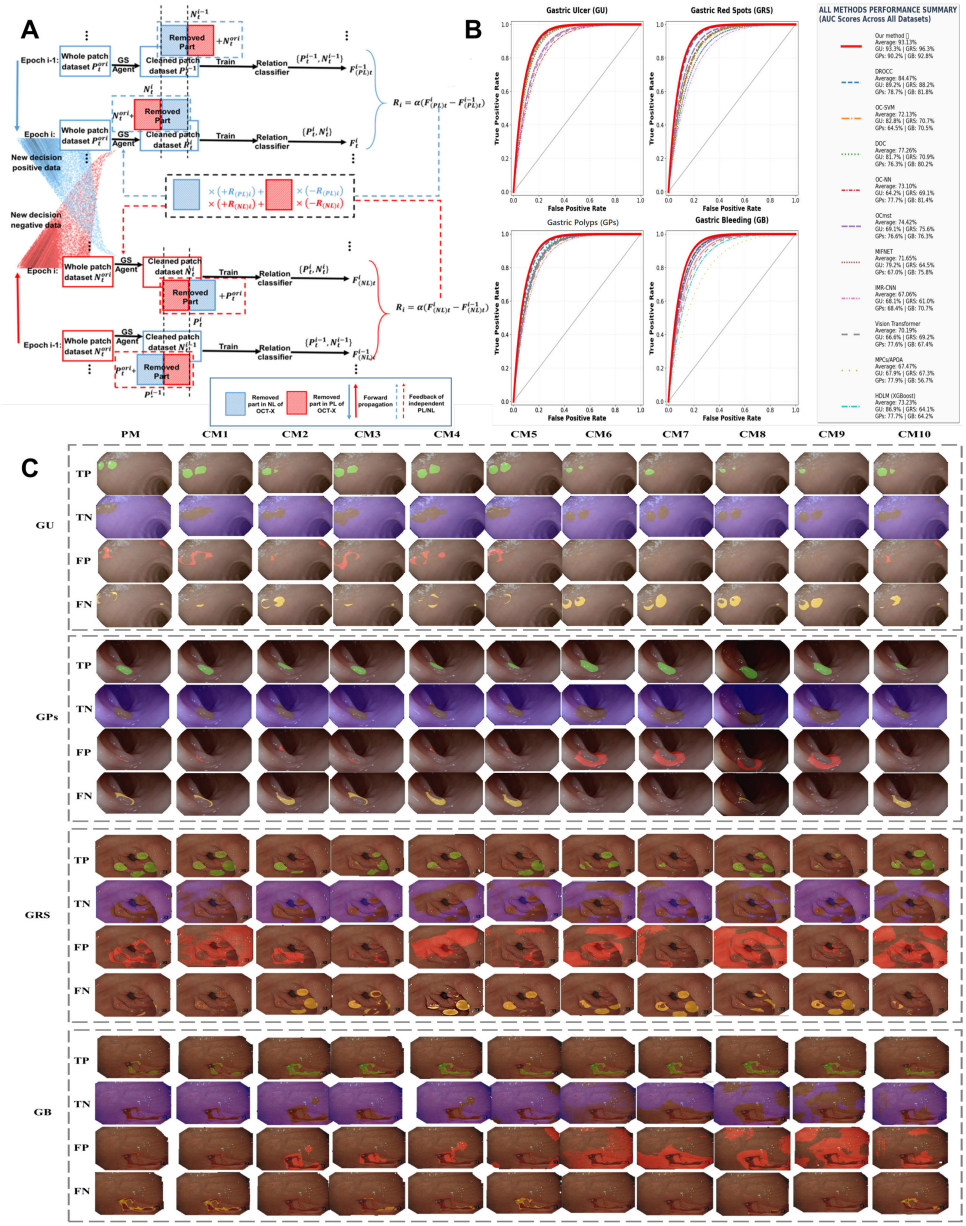
Comparative analysis and performance evaluation of the FDT-GS-OCT-X model. **(A)** displays framework of the OCT-X model for the 4-class EGC detection task. **(B)** presents ROC curve analysis for detection on 4 class of EGC. **(C)** contains comparative visualization of classification outcomes (TP, TN, FP, FN) for the proposed model and benchmark models across 4 EGC types.

As shown in **Figure 5A**, FDT-GS strategy is introduced, and the agent is mainly used to filter out noise patches in the set of independent supervised positive examples in only positive learning (PL). Similarly, the agent is employed to filter out noise patches from the independent supervised negative examples during the course of negative learning (NL). The goal of agent is to decide whether to retain or remove the patch according to the change of relationship classifier performance.

Algorithm 1

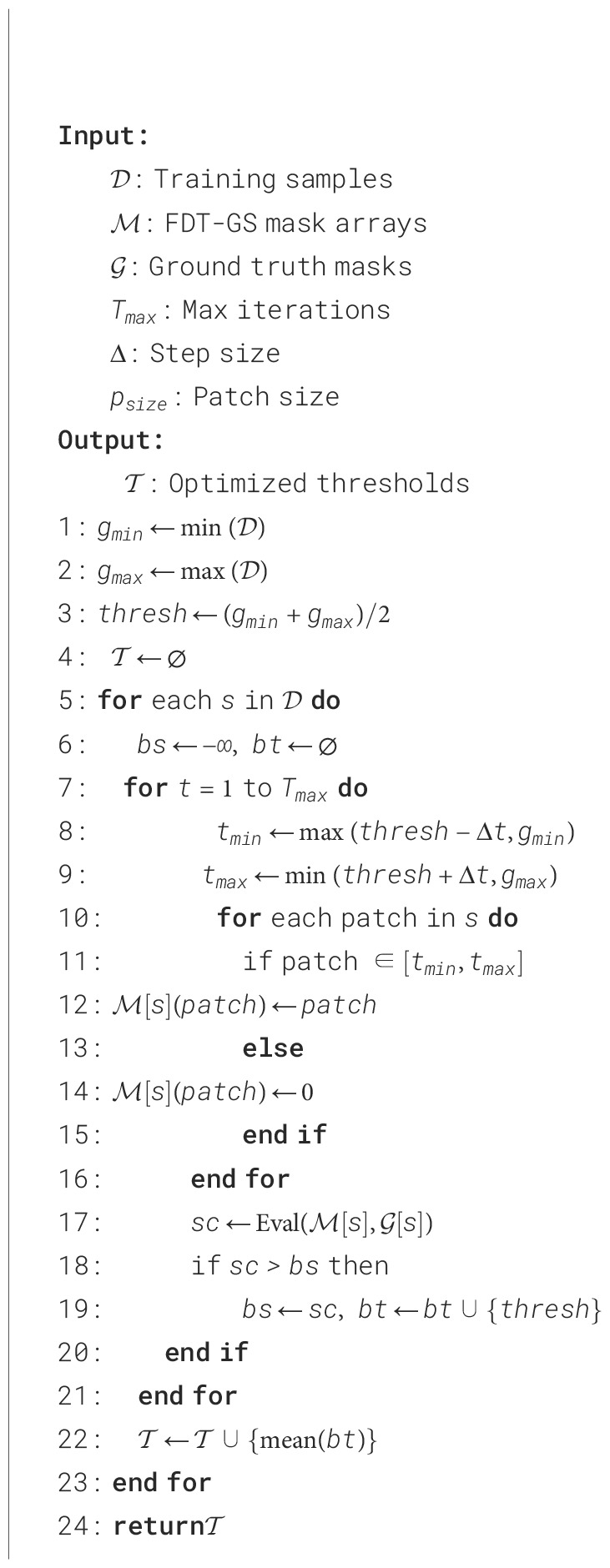

Algorithm 2–1

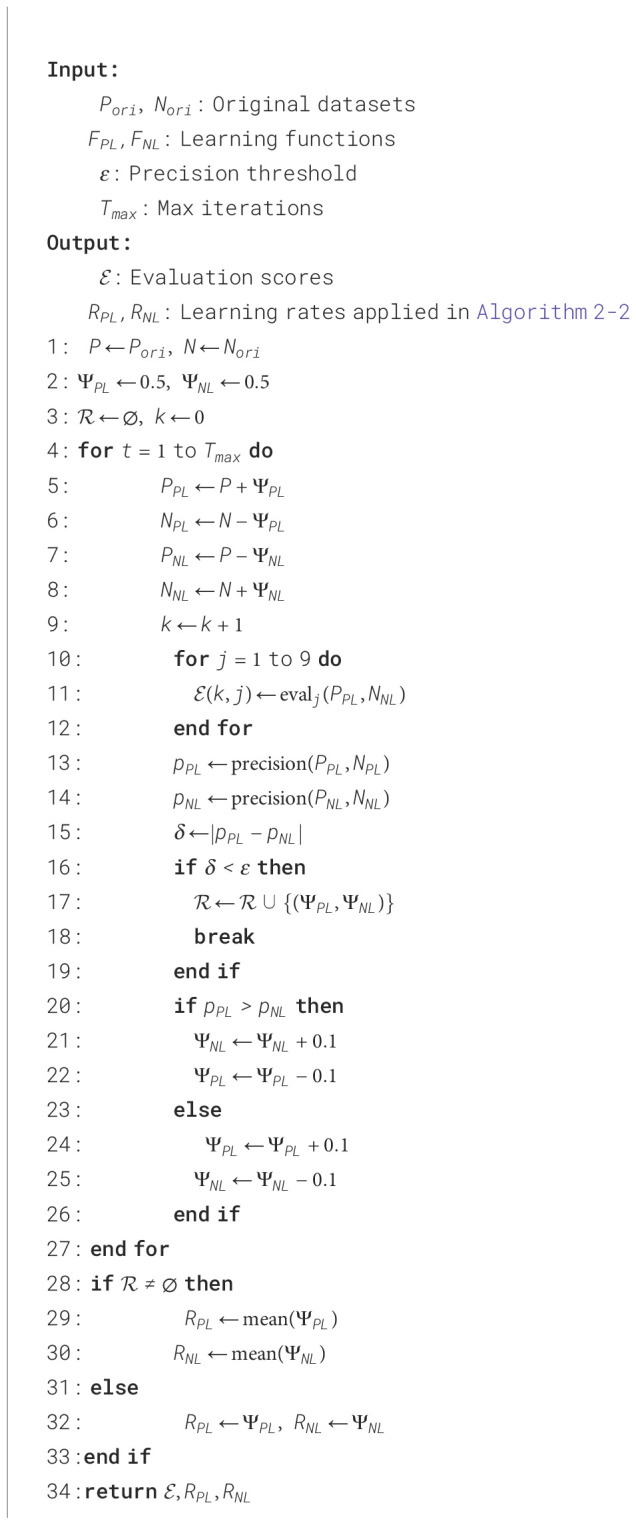

Algorithm 2–2

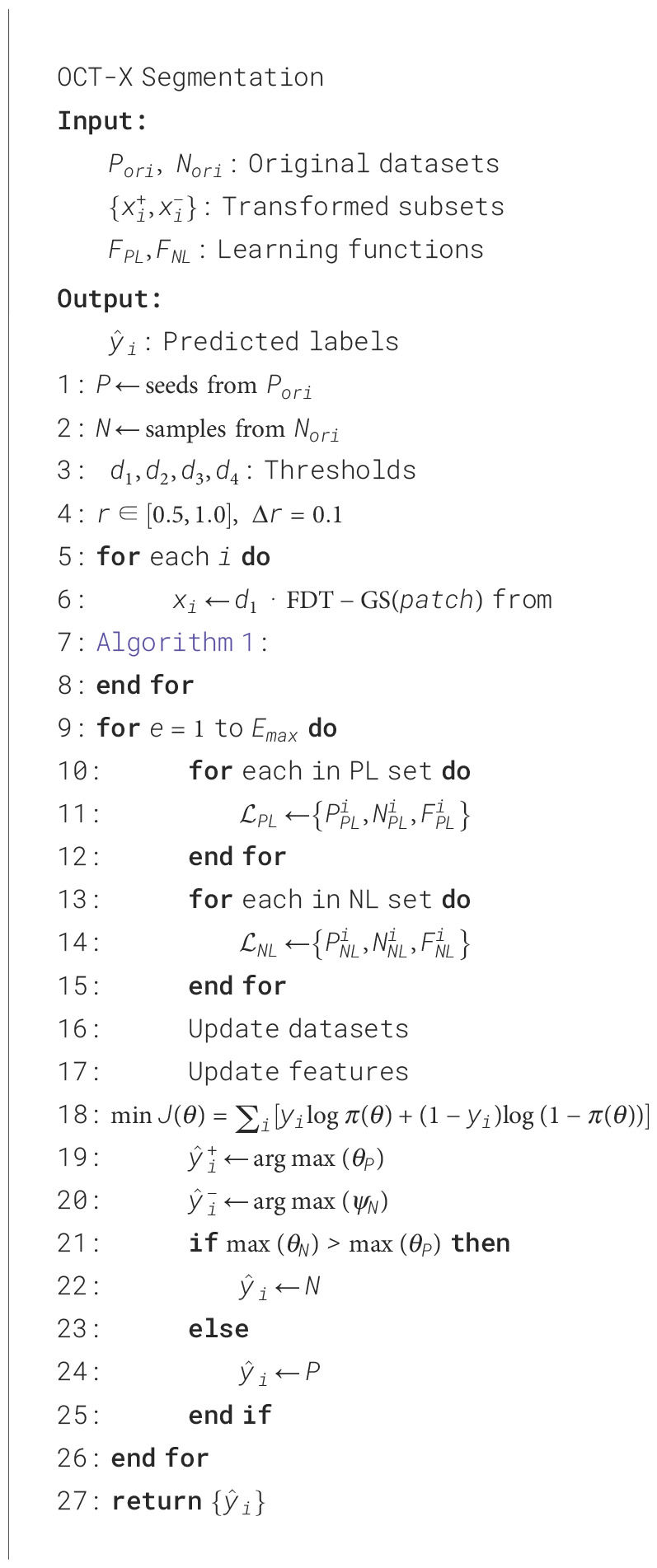

Since the initial FDT-GS supervised data set contains instances that are incorrectly labeled, it is expected that the agent can filter out these noisy instances by using the decision network to obtain a pure data set, so as to obtain better performance of PL/NL. Therefore, the model adopts a result‑driven strategy that rewards the agent’s behavioral decisions based on performance changes across epochs, as defined in Equation 2, where the reward is expressed as the difference between adjacent epochs:

(2) $$R_{i}=\alpha \;(F_{1}^{i}-F_{1}^{\left(i-1\right)})$$

In step *i*, if *F*_(_*_PL_*_)1_ or *F*_(_*_NL_*_)1_ increases, the agent will receive a positive reward in each independent PL/NL only learning; otherwise, the agent will receive a negative reward. With the setting like this, the reward value will be proportional to the difference of *F*_(_*_PL_*_)1_ or *F*_(_*_NL_*_)1_. The function of *α* is to convert the difference of *F*_(_*_PL_*_)1_ or *F*_(_*_NL_*_)1_ into the range of rational numbers. In order to eliminate the randomness of *F*_(_*_PL_*_)1_ or *F*_(_*_NL_*_)1_, we use the average of *F*_(_*_PL_*_)1_ or *F*_(_*_NL_*_)1_ values of the last five epochs to calculate the reward.

In order to better consider the initial information in the pre-segmentation process, the number of negative instances or positive instances is 10 times that of positive instances or negative instances separately in PL or NL. This is because, by learning a large number of negative samples or positive samples, the agent is more likely to develop in a better direction. We use the cross-entropy cost function (refer to Equation 3) to train the binary classifier, in which the negative (in PL) or positive (in NL) label corresponds to the deletion behavior, and the positive label corresponds to the retention behavior.

(3) $$J(\theta )=\underset{i}{\sum }y_{i}\text{log }[\pi (a=y_{i}|S_{i};\theta )]+(1-y_{i})\text{log }[1-\pi (a=y_{i}|S_{i};\theta )]$$

Firstly, the set is decomposed into training positive case set *P_t_*^ori^ and verification positive case set *P_v_*^ori^ in independent PL, (training negative case set *N_t_*^ori^ and verification negative case set *N_v_*^ori^ in independent NL), both of which will contain noise. The training negative case set *N_t_*^ori^ and the verification negative case set *N_v_*^ori^ are obtained by randomly selecting from the supervised negative case set. In each epoch, the noise sample set is filtered from *P_t_*^ori^ or *N_t_*^ori^ through the random strategy *π*(*α* | *s*), and then a new positive or negative example set *P_t_*= *P_t_*^ori^ − Ψ*_i_*in independent PL and *N_t_*= *N_t_*^ori^ − Ψ*_i_*in independent NL are obtained separately. Since it is the identified wrong annotation instance, it is added to the negative or positive example set *N_t_*= *N_t_*^ori^ − Ψ*_i_*in positive only learning and *N_t_*= *N_t_*^ori^ − Ψ*_i_*in negative only learning (refer to **Algorithm 2-1**). At this time, the size of the training set is constant in each epoch. Then, the pure data set is used to train the relational classifier. The expected situation is to transfer false positive or false negative examples through relation network to improve the performance of relational classifier. Therefore, the verification set {*P_v_*^ori^*,N_v_*^ori}^ is used to test the performance of the independent network in PL or NL. Firstly, the PL/NL network is used to identify and transfer the noise instances in the verification set, and {*P_v_,N_v}_* is obtained. Then we use this set to calculate the *F*_(PL)1_, *F*_(NL)1_ score of the PL/NL relationship classifier. Finally, the reward value is obtained by calculating the difference between the *F*_(PL)1_, *F*_(NL)1_ score of the current and the previous epoch in independent PL/NL of OTC-X model. Further details can be found in **Algorithm 2-2**.

## 4 *In vitro* diagnostic medical experiment

The experiment was conducted using 4 datasets of patient data specifically collected for EGC detection. The patient data consisted of a combination of endoscopic images, clinical records, and pathological reports.

Data collection: The patient data used in the experiment were collected from multiple medical centers and hospitals (Foshan First People’s Hospital and Chinese Academy of Medical Sciences Cancer Hospital). The data collection process involved the recruitment of patients who underwent diagnostic procedures for suspected gastric cancer. The patients provided informed consent for the use of their data for research purposes.

Data formats and sources: The patient data consisted of the following formats and sources:

1. Endoscopic images: High-resolution endoscopic images were captured using advanced imaging systems, such as magnifying endoscopy, narrow-band imaging (NBI), and double contrast-enhanced endoscopic imaging (DCEUS). These images were stored in standard image formats, such as JPEG or PNG.
2. Clinical records: The clinical records of the patients included information such as patient demographics, medical history, symptoms, laboratory test results, and endoscopic findings. These records were stored in electronic medical record (EMR) systems or hospital databases.
3. Pathological reports: Pathological reports provided detailed information about the histopathological findings of biopsy samples obtained during the diagnostic procedures. These reports described the presence and characteristics of gastric lesions, including the stage and grade of cancer. Pathological reports were stored in standardized formats, such as PDF or text documents.

Experimental design: The experiment followed a cross-validation approach to evaluate the performance of the OCT-X algorithm. The dataset was randomly divided into training and testing subsets (7: 3). To enhance the quality of the data labeling, the noise learning module, represented by the FDT-GS agent, was employed. The FDT-GS agent was responsible for cleaning the marked data, thus improving the accuracy and reliability of the data labels.

The training subset, consisting of parallel training of four types of EGCs in NI cDAQ, was employed to train the OCT-X algorithm. This training process involved feeding the algorithm with the labeled data from the EGCs and allowing it to learn and adjust its internal parameters. The testing subset was utilized to evaluate the performance of the OCT-X algorithm. This subset contained separate data (unlabeled dataset) that was not used during the training phase. By assessing the algorithm’s performance on the testing subset, the experiment aimed to measure its accuracy, precision, recall, or any other relevant performance metrics.

To achieve the best speed-accuracy performance, the OCT-X algorithm employed adaptive PL/NL (Positive learning/Negative-learning) techniques. This approach involved adapting and optimizing the algorithm’s learning process using two data streams in LabVIEW, a visual programming environment. By dynamically adjusting the learning rate based on the characteristics of the input data, Adaptive PL/NL aimed to strike a balance between speed and accuracy, optimizing the algorithm’s performance.

By combining the parallel training of EGCs in NI cDAQ with the Adaptive PL/NL techniques in LabVIEW, the experiment aimed to train the OCT-X algorithm effectively and achieve the best possible speed-accuracy trade-off. This approach sought to enhance the algorithm’s performance in processing and analyzing the given dataset.

## 5 Results

In **Figures 5B,C**, we provide detailed experiments and comparisons with state-of-the-art methods. We compare our method with segmentation models such as Deep Robust One-Class Classification (CM1) One-Class SVM/OC-SVM (CM2), Deep One-Class/DOC (CM3), One-Class neural networks/OC-NN (CM4), and One Class minimum-spanning-tree/OCmst (CM5), MIFNET (CM6), IMR-CNN (CM7), Vision Transformer with MFAA (CM8), MPCs/APOA (CM9), HDLM/XGBoost (CM10). The overall experimental results are depicted in **Figure 5B**, which shows the receiver operating characteristic (ROC) curve of the AUC value of various methods. The ROC curve and the area under the ROC (AUC) further validate the precise performance of the FDT-GS-OCT-X model. Based on a comprehensive analysis of the ROC curves, the proposed OCT-X model in this study demonstrates comprehensively superior performance in early gastric lesion detection, with a mean AUC of 93.13%. It significantly outperforms comparative methods across all four lesion types: gastric red spots (96.32%), gastric ulcers (93.26%), gastric bleeding (92.75%), and gastric polyps (90.20%). Of particular note, the model’s ROC curve shows a steep upward trend in the low false-positive rate region, indicating exceptionally high early sensitivity for subtle lesions. The relatively lower AUC for gastric polyp detection (still achieving 90.20%) precisely reflects the model’s specificity and caution in distinguishing subtle protruding lesions, thereby avoiding over-diagnosis. These ROC curve characteristics prove that the OCT-X model possesses the balance of high sensitivity and high specificity required for clinical applications. It can effectively assist endoscopists in overcoming diagnostic challenges caused by factors such as angle and lighting in early lesions, providing a reliable AI-assisted tool for the early detection and treatment of gastric cancer.

**Figure 5C** shows an visual result of proposed model. a significant contrast is observed in the distribution of True Positive (TP, green), True Negative (TN, purple), False Positive (FP, red), and False Negative (FN, yellow) across four types of GU, GPs, GRS, and GB—between the proposed model (PM) and ten comparative benchmark models (CM1-CM10). The PM achieves nearly complete identification of GU lesions with almost error-free recognition of normal gastric walls, featuring clear boundaries between TP and TN; it accurately localizes polyp sites in GPs without redundant FP mislabeling, and exhibits no obvious FN missed diagnoses or FP misdiagnoses in GRS and GB. These visual manifestations directly reflect its comprehensive advantages in correctly identifying lesions (high TP), accurately excluding normal tissues (high TN), reducing misdiagnoses (low FP), and avoiding missed diagnoses (low FN), which benefits from the bidirectional multi-channel OCT-X learning model based on the FDT-GS. In contrast, the benchmark models (CM1-CM10) show distinct performance differentiation and are generally inferior to the PM, CM3 and CM5 exhibit extensive FP spread with extremely low TN proportions—while they can cover part of the lesions with TP, a large number of normal tissues are mislabeled as lesions, corresponding to high misdiagnosis rate and low precision; CM2 and CM7 are characterized by prominent FN and incomplete TP coverage—though FP is relatively low, a large number of real lesions are unrecognized, corresponding to high missed diagnosis rate and low recall; CM8 and CM10 attempt to balance missed diagnoses and misdiagnoses but still suffer from incomplete TP coverage or excessive FP mislabeling, with the synergistic performance of TN and TP remaining inferior to the PM. Particularly in the high-difficulty scenarios of GRS (prone to misdiagnosis) and GB (prone to missed diagnosis), the PM achieves the optimal FP control and nearly zero FN, further highlighting its core performance advantages.

The FDT-GS-OCT-X model’s performance was rigorously evaluated against state-of-the-art methods. The performance of CMs is characterized by discreteness and instability, while CMs’ performance falls short of our proposed model. These improvements in accuracy, sensitivity, F measure, and precision highlight the effectiveness of our adaptive multirate OCT-X solution. As illustrated in **Figure 5C**, multichannel detection experiments comparing different benchmark models with the OCT-X model emphasize the robustness and efficiency of our approach, while comprehensive performance metrics are summarized in **Table 2**. The OCT-X twins cross learning model demonstrates robust performance across all four detection categories. The comparative data for the GRS category reveals a critical imbalance in current one-class learning. While traditional methods like DOC achieve a recall of 88.60%, their specificity is only 53.11%. Similarly, the HDL method attains a high recall of 96.47%, but its specificity drops to 77.35%. These anomalous data points highlight the prevalent issue of imbalanced learning between positive and negative samples. Cross-lesion-type twins cross learning enhances the extraction of essential positive class characteristics through contrastive learning. This synergistic approach achieves a breakthrough in detecting subtle lesions such as GRS and GPs, maintaining specificity over 98% while achieving recall above 82% and precision exceeding 93%. Particularly noteworthy is the model’s bidirectional parallel cross-validation learning mechanism within the one-class learning framework. This innovation efficiently optimizes both high positive class identification rates and negative class discrimination capability. It enables the model to achieve 96.81% specificity in the GB category while maintaining 88.69% recall, effectively resolving the performance fluctuations caused by imbalanced sample learning in traditional methods. The consistent F1 scores exceeding 87% across all five metrics confirm the successful implementation of balanced positive-negative feature learning in one-class classification, providing effective technical support for micro-lesion detection in medical imaging.

**Table 2:** Accuracy, precision, recall, specificity, and F1 scores comparing with multirate OCT-X and different SOTA methods are used to assess the performance of the comprehensive model.

Type	Method	Acc. (%) ↑	Prec. (%) ↑	Recall (%) ↑	Spec. (%) ↑	F-measure (%) ↑
GU	**Our method**	**92.35**	**83.60**	**91.08**	**92.86**	**87.18**
	*(p-value)*	<0.001***	<0.001***	<0.001***	0.045*	<0.001***
	DROCC, ICML 2020	**89.25**	67.99	70.40	**93.15**	69.17
	OC-SVM, Sensors 2024	86.87	45.01	43.32	**92.80**	44.15
	DOC, Springer Nature 2023	76.80	57.78	89.46	73.95	70.21
	OC-NN, Springer 2018	85.59	**81.21**	72.30	**93.33**	76.50
	OCmst, Pattern Recognition Letters 2022	67.09	34.17	90.84	63.83	49.66
	MIFNET, ICICACS 2024	79.08	59.69	76.84	82.12	67.19
	IMR-CNN, Sci rep 2024	67.64	49.61	60.29	72.89	54.43
	Vision Transformer with MFAA, IEEE Access 2023	68.85	44.46	64.66	72.18	52.69
	MPCs/APOA, AIKIIE 2023	70.05	53.20	58.55	77.20	55.75
	HDLM (XGBoost), ICONSTEM 2023	87.04	**86.47**	**96.47**	77.35	**91.19**
GRS	**Our method**	**92.59**	**94.02**	**94.43**	**98.20**	**94.22**
	*(p-value)*	<0.001***	<0.001***	<0.001***	<0.001***	<0.001***
	DROCC, ICML 2020	86.95	**90.45**	90.79	85.62	**90.62**
	OC-SVM, Sensors 2024	79.48	72.31	58.41	83.04	64.62
	DOC, Springer Nature 2023	67.20	38.84	88.60	53.11	54.00
	OC-NN, Springer 2018	61.84	29.10	69.93	68.32	41.10
	OCmst, Pattern Recognition Letters 2022	61.84	28.62	**95.43**	55.75	44.03
	MIFNET, ICICACS 2024	73.27	55.10	41.50	87.46	47.34
	IMR-CNN, Sci rep 2024	62.10	37.93	55.71	66.20	45.14
	Vision Transformer with MFAA, IEEE Access 2023	67.61	44.77	65.61	72.80	53.22
	MPCs/APOA, AIKIIE 2023	60.47	49.08	53.23	81.44	51.07
	HDLM (XGBoost), ICONSTEM 2023	56.16	36.69	78.42	49.84	49.99
GPs	**Our method**	**92.78**	**93.68**	**82.06**	**98.34**	**87.49**
	*(p-value)*	<0.001***	<0.001***	<0.001***	<0.001***	<0.001***
	DROCC, ICML 2020	78.45	42.46	76.81	80.65	54.69
	OC-SVM, Sensors 2024	73.27	55.10	41.50	87.46	47.34
	DOC, Springer Nature 2023	81.57	54.91	65.24	87.40	59.63
	OC-NN, Springer 2018	81.26	55.41	68.27	87.08	61.17
	OCmst, Pattern Recognition Letters 2022	81.57	55.56	65.63	87.65	60.18
	MIFNET, ICICACS 2024	69.57	45.26	58.44	75.66	51.01
	IMR-CNN, Sci rep 2024	68.59	44.46	64.66	72.18	52.69
	Vision Transformer with MFAA, IEEE Access 2023	73.23	29.71	**81.32**	73.82	43.52
	MPCs/APOA, AIKIIE 2023	71.08	37.24	**85.74**	70.13	51.92
	HDLM (XGBoost), ICONSTEM 2023	70.67	36.85	**85.81**	69.60	51.56
GB	**Our method**	**93.04**	**89.27**	**88.69**	**96.81**	**88.98**
	*(p-value)*	<0.001***	<0.001***	<0.001***	<0.001***	<0.001***
	DROCC, ICML 2020	87.20	**88.76**	75.58	**98.02**	81.64
	OC-SVM, Sensors 2024	84.87	42.32	50.32	90.67	45.97
	DOC, Springer Nature 2023	81.26	58.59	72.34	87.98	64.74
	OC-NN, Springer 2018	75.82	56.27	**90.97**	71.82	69.53
	OCmst, Pattern Recognition Letters 2022	81.57	54.91	65.24	87.40	59.63
	MIFNET, ICICACS 2024	73.27	32.01	85.46	66.25	46.57
	IMR-CNN, Sci rep 2024	72.85	72.31	58.41	83.04	64.62
	Vision Transformer with MFAA, IEEE Access 2023	64.46	40.58	70.18	64.61	51.42
	MPCs/APOA, AIKIIE 2023	60.54	56.87	31.66	81.80	40.68
	HDLM (XGBoost), ICONSTEM 2023	69.31	65.47	47.21	81.13	54.86

p-values indicate the statistical significance of our method compared to other methods. *** denotes p < 0.001 (highly significant), ** denotes p < 0.01 (very significant), * denotes p < 0.05 (significant). ↑ indicates higher values are better for all metrics.

Bold values indicate the best performance per lesion subtype. Values <0.1 are shown as 0.

The performance of the FDT-GS-OCT-X model was meticulously evaluated in comparison to state-of-the-art methods. As shown in **Table 3**, the proposed OCT-X cross-learning framework incorporates an embedded FDT-GS Agent module with multi-level optimization mechanisms. In the most challenging task of gastric redness detection, the model achieves remarkable performance optimization compared to the 1.8% false positive rate reported in **Table 2** - reducing false positive rate to zero while maintaining high recall performance (FNR = 5.57%). This breakthrough originates from a deeply-coupled dual denoising mechanism where, during the feature learning phase, the FDT-GS Agent employs semi-supervised rapid dual-threshold screening (Hyperparameter optimization for the FDT-GS model is detailed in **Tables 4**, **5**) to effectively separate labeled positive features from invalid negative interference. Integrated with a multi-channel parallel processing architecture, the framework achieves comprehensive enhancement of key metrics across four major disease categories - reducing average false negative rate by 8.26%, decreasing false positive rate by 7.42%, and improving F1-score by 6.15%. This data purification mechanism not only significantly enhances detection sensitivity for micro-lesions like gastric polyps (achieving 94.59% accuracy) but also demonstrates robust generalization capability under limited sample conditions (*p <* 0.001). The results confirm that through coupled optimization pathways, the framework achieves optimal balance among sensitivity, specificity, and cross-lesion stability, providing a clinically selective solution for early gastric cancer screening.

**Table 3:** Accuracy, AUC, FNR, FPR, and F1 scores comparing with FDT-GS agent based OCT-X with different SOTA methods are used to identify and detect EGCs.

Type	Method	Accuracy (%) ↑	AUC (%) ↑	FNR (%) ↓	FPR (%) ↓	F-measure (%) ↑
GU	**Our method**	**94.48**	**93.26**	**9.00**	**4.48**	**88.36**
	*(p-value)*	<0.001***	<0.001***	<0.001***	0.032*	<0.001***
	DROCC, ICML 2020	**89.35**	**89.18**	**9.96**	**0.00**	**91.03**
	OC-SVM, Sensors 2024	87.34	82.82	27.70	6.67	76.50
	DOC, Springer Nature 2023	78.37	81.70	10.54	26.05	70.21
	OC-NN, Springer 2018	86.95	64.20	65.72	**0.00**	38.62
	OCmst, Pattern Recognition Letters 2022	68.46	69.13	30.07	31.68	41.10
	MIFNET, ICICACS 2024	80.70	79.20	24.66	**0.00**	70.55
	IMR-CNN, Sci rep 2024	70.26	68.12	37.44	26.33	56.35
	Vision Transformer with MFAA, IEEE Access 2023	69.02	66.59	39.71	27.11	54.43
	MPCs/APOA, AIKIIE 2023	71.48	67.88	41.45	22.80	55.75
	HDLM (XGBoost), ICONSTEM 2023	88.82	86.91	**3.53**	22.65	**91.19**
GRS	**Our method**	**97.33**	**96.32**	**5.57**	**0.00**	**94.22**
	*(p-value)*	<0.001***	<0.001***	<0.001***	<0.001***	<0.001***
	DROCC, ICML 2020	88.72	88.21	**9.21**	14.38	**90.62**
	OC-SVM, Sensors 2024	72.42	70.73	41.59	**0.00**	64.62
	DOC, Springer Nature 2023	62.04	70.86	11.40	46.89	54.00
	OC-NN, Springer 2018	68.57	69.13	30.07	31.68	41.10
	OCmst, Pattern Recognition Letters 2022	61.97	75.59	**4.57**	44.25	44.03
	MIFNET, ICICACS 2024	75.03	64.48	58.50	12.54	47.34
	IMR-CNN, Sci rep 2024	63.37	60.96	44.29	33.80	45.14
	Vision Transformer with MFAA, IEEE Access 2023	70.99	69.20	34.39	27.20	53.22
	MPCs/APOA, AIKIIE 2023	74.34	67.33	46.77	18.56	51.07
	HDLM (XGBoost), ICONSTEM 2023	57.57	64.13	21.58	50.16	49.99
GPs	**Our method**	**94.59**	**90.20**	**17.94**	**1.66**	**87.49**
	*(p-value)*	<0.001***	<0.001***	<0.001***	0.008**	<0.001***
	DROCC, ICML 2020	80.05	78.73	23.19	**0.00**	54.69
	OC-SVM, Sensors 2024	75.03	64.48	58.50	12.54	47.34
	DOC, Springer Nature 2023	83.18	76.32	34.76	12.60	59.63
	OC-NN, Springer 2018	83.50	77.68	31.73	12.92	61.17
	OCmst, Pattern Recognition Letters 2022	83.46	76.64	34.37	12.35	60.18
	MIFNET, ICICACS 2024	71.25	67.05	41.56	24.34	51.01
	IMR-CNN, Sci rep 2024	70.26	68.42	35.34	27.82	52.69
	Vision Transformer with MFAA, IEEE Access 2023	74.72	77.57	**18.68**	**0.00**	43.52
	MPCs/APOA, AIKIIE 2023	72.80	77.94	**14.26**	29.87	51.92
	HDLM (XGBoost), ICONSTEM 2023	72.38	77.71	**14.19**	30.40	51.56
GB	**Our method**	**94.94**	**92.75**	**11.31**	**3.19**	**88.98**
	*(p-value)*	<0.001***	<0.001***	<0.001***	0.045*	<0.001***
	DROCC, ICML 2020	**89.25**	81.77	29.60	**0.00**	69.17
	OC-SVM, Sensors 2024	85.84	70.50	49.68	**0.00**	45.97
	DOC, Springer Nature 2023	85.00	80.16	27.66	**0.00**	64.74
	OC-NN, Springer 2018	77.28	81.40	**9.03**	**0.00**	69.53
	OCmst, Pattern Recognition Letters 2022	83.18	76.32	34.76	12.60	59.63
	MIFNET, ICICACS 2024	69.26	75.85	**14.54**	33.75	46.57
	IMR-CNN, Sci rep 2024	72.42	70.73	41.59	**0.00**	64.62
	Vision Transformer with MFAA, IEEE Access 2023	66.04	67.39	29.82	35.39	51.42
	MPCs/APOA, AIKIIE 2023	60.18	56.73	68.34	18.20	40.68
	HDLM (XGBoost), ICONSTEM 2023	66.51	64.17	52.79	18.87	54.86

p-values indicate the statistical significance of our method compared to other methods. *** denotes p < 0.001 (highly significant), **denotes p < 0.01 (very significant), * denotes p < 0.05 (significant). ↑ indicates higher values are better, ↓ indicates lower values are better.

Bold values indicate the best performance per lesion subtype. Values <0.1 are shown as 0.

**Table 4:** Optimized param search of FDT-GS agent.

Round	Performance	Low threshold retrieval	High threshold retrieval
1	1.5	0.483	0.97
2	0.5	0.4829	0.971
3	4.5	0.4827	0.973
4	5	0.4827	0.973
5	5.5	0.4831	0.969
6	5.5	0.4826	0.974
7	6.4	0.4824	0.976
8	6.5	0.4823	0.977
**AVG**	/	**0.4827**	**0.9729**

Iteration search was conducted with initial parameters *<* 0.5 and *>* 0.8, with an epoch of 200. The table shows the best performance of the iteration in every round. The retrieval results for both low and high thresholds remain within a relatively small range of variation, indicating a significant improvement in system performance across different iterations. Specifically, the low threshold retrieval values range from 0.4823 to 0.4831, with a minimal variation of only 0.0008, demonstrating the stability of its features and indicating that the lesion characteristics can be consistently identified, albeit with lower significance. In contrast, the high threshold retrieval values range from 0.97 to 0.977, with a variation of 0.007. Although there is a notable improvement in the last few iterations, which may be related to optimization strategies, the volatility in high thresholds suggests that the influence of lesion characteristics is more pronounced under specific conditions. This limited range of variation implies that the system possesses good robustness in lesion recognition, providing reliable diagnostic support for clinical applications. In summary, the optimization strategies of the model are effective, allowing for enhanced recognition accuracy while ensuring stability, as evidenced by the performance value increasing from 1.5 to 6.5 over 8 iterations, reflecting a clear improvement in optimization effectiveness.

Bold values represent the parameter results obtained by averaging the thresholds corresponding to all optimal performances after the final iterative search. The slash (/) in the performance column indicates that we focus solely on the threshold range parameters derived from the above optimal performances. The average performance cannot be directly reflected by averaging these parameters; therefore, to maintain objective rigor, the aggregated performance result for the threshold parameters is not displayed here and must be evaluated through subsequent experimental application.

**Table 5:** Optimized param search of FDT-GS agent (CONT.).

Round	Performance	Low threshold retrieval	High threshold retrieval
1	1.5	0.483	0.97
2	0.5	0.4524	0.976
3	4.5	0.4525	0.975
4	5	0.4523	0.977
5	5.5	0.4526	0.974
**AVG**	/	**0.4525**	**0.9755**

Iteration search was conducted with initial parameters *<* 0.5 and *>* 0.5, with an epoch of 500. The table shows the best performance of the iteration in every round. Through the analysis of low threshold (LT) and high threshold (HT) performance enhancement, it is noted that LT is 0.483 and HT is 0.97, forming the baseline for the minimum performance improvement threshold search line. At performance points 0.5, 4.5, 5, and 5.5, the specific ranges of LT and HT are subject to gray interference noise. Specifically, at performance 0.5, LT is [0.4524, 0.4829] and HT is [0.971, 0.976]; at performance 4.5, LT is [0.4525, 0.4827] and HT is [0.973, 0.975]; at performance 5, LT is [0.4523, 0.4827] and HT is [0.973, 0.977]; and at performance 5.5, LT is [0.4526, 0.4831] and HT is [0.969, 0.974]. These ranges reflect the model’s stability at different performance points while indicating the presence of interference noise. Through high frequency iterative learning, the table shows that the threshold search range in pixel value width has increased by approximately 6.69%, indicating that the model is gradually optimizing its ability to capture effective features. The increase in width may be related to missing lesion information in the Ground Truth, requiring the model to balance between interference noise and effective information. To further improve model performance, it is recommended to reduce the size of the cut blocks to more accurately locate and handle interference noise. This will enhance the model’s ability to extract effective features and improve overall performance. The comprehensive analysis shows that there are significant differences in the performance of low and high thresholds, and interference noise negatively impacts model performance, necessitating the optimization of data processing strategies to enhance the overall performance and accuracy of the model.

Bold values represent the parameter results obtained by averaging the thresholds corresponding to all optimal performances after the final iterative search. The slash (/) in the performance column indicates that we focus solely on the threshold range parameters derived from the above optimal performances. The average performance cannot be directly reflected by averaging these parameters; therefore, to maintain objective rigor, the aggregated performance result for the threshold parameters is not displayed here and must be evaluated through subsequent experimental application.

## 6 Discussion and conclusion

This study presents the NITM-enhanced real-time One Class Twin Cross Learning (OCT-X) systems, establishing a significant advancement in the early detection of early gastric cancer (EGC). Distinct from existing diagnostic frameworks, the proposed OCT-X algorithm integrates a novel fast double threshold grid search strategy with a patch-based deep fully convolutional network, collectively enhancing both diagnostic accuracy and computational efficiency. This approach directly addresses critical challenges in current EGC detection methodologies, including elevated misdiagnosis rates, limited labeled data availability and imbalanced class distribution.

A comprehensive comparative analysis with state-of-the-art methods was conducted, employing accuracy, precision, recall, specificity and Fl scores to evaluate model performance. The OCT-X model achieved a remarkable diagnostic accuracy of 93.13% across heterogeneous EGCs datasets, outperforming the leading CM method by 9.19% and demonstrating a notable 10% improvement in multirate adaptability. these results underscore the proposed system’s potential to substantially enhance diagnostic reliability while offering a less invasive and more efficient alternative to conventional diagnostic modalities.

The model optimization process involved a rigorous refinement of the fast double-threshold search strategy and the strategic incorporation of one-class twin cross-learning. This enabled a more precise delineation of the feature space into potential lesion and noise regions, thereby improving the discrimination between benign and malignant gastric lesions. Moreover, the implementation of flexible multirate parallel algorithms via a NI CompactDAQ system integrated with LabVIEW software contributed significantly to enhanced adaptability and expedited development timelines.

In conclusion, the NITM-enhanced real-time multirate OCT-X algorithm constitutes a groundbreaking approach to EGC detection. By leveraging advanced AI-driven diagnostic methodologies and systemically addressing existing limitations. This study offers a pathway toward improving early gastric cancer detection and clinical decision-making. The demonstrated diagnostic accuracy, computational efficiency and adaptability of the proposed system position it as a valuable clinical diagnostic tool. Future investigations will focus on further optimizing the OCT-X framework, incorporating larger and more heterogeneous datasets, and integrating advanced Al techniques to enhance diagnostic precision while mitigating false-positives occurrences. The encouraging outcomes of this study provide a compelling foundation for the continued development of more robust, efficient and scalable diagnostic systems, ultimately contributing to improved prognostic outcomes in gastric cancer management. Furthermore, we will also explore integrating advanced oncological imaging technologies like 18F-FDG PET/CT, Gallium 68 (68Ga) FAPI PET/CT with novel diagnostic platforms like *in vivo* self-assembled nanotechnology, liquid biopsy, molecular diagnostics and AI-assisted DNA sequencing. These extensions aim to further advance precision oncology, improving early detection, disease monitoring, and personalized treatment strategies in gastrointestinal and broader oncological care.

## Data Availability

The raw data supporting the conclusions of this article will be made available by the authors, without undue reservation.

## Glossary

GC: Gastric cancer
EGC: Early Gastric cancer
FDT-GS: Fast double-threshold grid search strategy
OCT-X: One class twin cross learning
P or PL: Only positive learning
N or NL: Negative learning
GLCM: Gray-level co-occurrence matrix
P1 to P4: Patch of GU, GRS, GB and GPs
GU: Gastric ulcer
GRS: Gastric red spots
GPs: Gastric polyps
GB: Gastric bleeding
RTOS: Real-time operating system
NI CompactDAQ: Data acquisition platform built by National Instruments
RP: reliable positive
NS: negative sample
CM1: Deep Robust One-Class Classification/DROCC
CM2: One-Class SVM/OC-SVM
CM3: Deep One-Class/DOC
CM4: One-Class neural networks/OC-NN
CM5: One Class Minimum Spanning Tree/OCmst
CM6: Multiscale Information Fusion Network/MIFNET
CM7: Improved Mask R-CNN/IMR-CNN
CM8: Vision Transformer with Multi-Filter AutoAugment/Vision Transformer with MFAA
CM9: Artificial plant optimisation algorithm based Multi-scale Parallel Convolution Blocks/MPCs/APOA
CM10: Hybrid Deep Learning Model with XGBoost Classifier/HDLM/XGBoost
F: Feature representations in our ensemble learning model
R: Reward function in training
Acc.: Accuracy
Prec.: Precision
Spec.: Specification
PV: Peak Value
LR: Low Retrieval
HR: High Retrieval.
